# Clinical and biochemical characteristics for patients with polyneuropathy, organomegaly, endocrinopathy, M-protein, and skin changes syndrome: a pilot observational study

**DOI:** 10.3389/fneur.2025.1528376

**Published:** 2025-02-04

**Authors:** Pei Li, Ye Zhang, Li-Min Luo, Wen-Qing Wang, Jing Li, Yan Cheng, Xiao Dang, Yang Chen, Wei Jiang

**Affiliations:** ^1^Department of Infectious Diseases, Tangdu Hospital, Fourth Military Medical University, Xi'an, Shaanxi, China; ^2^Department of Infectious Diseases, Air Force Hospital of Southern Theatre Command, Guangzhou, Guangdong, China; ^3^Department of Hematology, Tangdu Hospital, Fourth Military Medical University, Xi'an, Shaanxi, China; ^4^School of Basic Medicine, Fourth Military Medical University, Xi'an, Shaanxi, China; ^5^Department of Nursing Affairs, Tangdu Hospital, Fourth Military Medical University, Xi'an, Shaanxi, China

**Keywords:** POEMS syndrome, clinical characteristics, biochemical indicators, diagnosis, therapy

## Abstract

**Background:**

Polyneuropathy, organomegaly, endocrinopathy, M-protein, and skin changes (POEMS) syndrome is rare life-threatening condition associated with a clonal plasma cell neoplasm.

**Objective:**

The aim of this study is to investigate the clinical and biochemical features in patients with POEMS syndrome before and post-therapy.

**Methods:**

Characteristics of demographic information, underlying diseases, clinical manifestations, laboratory indicators, and imaging examination were retrospectively collected when diagnosed and post-therapy in the patients POEMS syndrome between 2018 and 2024.

**Results:**

Nineteen newly-diagnosed, treatment-naïve patients with POEMS syndrome were enrolled. The diagnosis of POEMS syndrome was re-analyzed and matched the diagnostic criteria updated in 2023. All patients presented the symptoms of polyneuropathy and positive for M-protein. Most patients suffered with hyperpigmentation (*n* = 18), organomegaly (*n* = 18), elevated vascular endothelial growth factor (VEGF) (*n* = 17), extravascular volume overload (*n* = 15), sclerotic bone lesions (*n* = 11), and hypothyroidism (*n* = 10). Serum alanine aminotransferase, aspartate aminotransferase, total bilirubin, total protein, and albumin levels were down-regulated, while uric acid level was up-regulated in patients with POEMS syndrome. Reduced triiodothyronine, thyroxine, free triiodothyronine levels were negatively correlated with urea nitrogen, creatinine, and uric acid levels in patients with POEMS syndrome. VEGF level, which was negatively correlated with Ca^2+^ level (*r* = −0.56), was reduced in most patients with POEMS syndrome receiving bortezomib/ixazomib and lenalidomide/thalidomide therapy. Aspartate aminotransferase, total protein, and estimated glomerular filtration rate levels were increased, while creatinine and uric acid levels were reduced post-therapy in patients with POEMS syndrome.

**Conclusion:**

Patients with POEMS syndrome had impaired liver and renal function, and effective therapy might partly repair the liver and renal dysfunction.

## Introduction

Polyneuropathy, organomegaly, endocrinopathy, M-protein, and skin changes (POEMS) syndrome is rare life-threatening condition associated with a clonal plasma cell neoplasm (1). The pathogenesis of POEMS syndrome is not fully elucidated. It is well-accepted that vascular endothelial growth factor (VEGF), a cytokine that secreted by different cell types (including plasma cells, marcophages, platelets, and osteoblasts, etc.) and targeting endothelial cells, correlates best with disease activity (1–3). VEGF overexpression appeared to be an important contributory element in POEMS syndrome (4), and the early evaluation of VEGF in inflammatory polyneuropathy could reduce the misdiagnosis of POEMS syndrome (5). However, anti-VEGF therapy fails to achieve successful results in POEMS syndrome (6). Although POEMS syndrome is a rare multisystemic plasma cell proliferation disorder (4), not all patients presents all aspects of the diagnostic criteria and the acronym does not encompass all manifestations of this syndrome (1). The prevalence of POEMS syndrome was reported as 0.3 cases per 100,000 individuals based on the Japanese national survey in 2003 (7). Importantly, POEMS syndrome has increasingly received attention due to the improved diagnostic level and quality of health services in China (8, 9).

Clinicians always overlook or misdiagnose at the early stage of POEMS syndrome due to the chronic disorders and multisystemic involvements, leading to delay in treatment and poor clinical outcomes. Approximate 31.9% patients inappropriately treated with glucocorticoids before the confirmation of POEMS syndrome (8). POEMS syndrome can masquerades or co-afflicts with numerous clinical conditions, such as diabetic foot (10), quasi-moyamoya disease (11, 12), hydrocephalus (13), portal hypertension (14, 15), porto-sinusoidal vascular disorder (16), chronic diarrhea (17), systemic mastocytosis (18), and heart failure (19, 20). Thus, it is pivotal to understand the clinical manifestations and routine laboratory indicators for early recognition of POEMS syndrome. Although biochemical characteristics, especially liver and renal functions, are not included in the diagnostic criteria for POEMS syndrome, several observational studies and reports have indicated the potential relationship between POEMS syndrome and hepatic/kidney dysfunctions (21–24). The renal function can be monitored by the determination of creatinine. The estimated glomerular filtration rate (eGFR) is usually calculated from equations by using serum creatinine level, age, race, sex, and body weight (25, 26). Accordingly, a comprehensive analysis of biochemical features of POEMS syndrome is required. Thus, we collected clinical presentations and laboratory/imaging data to improve the understanding of POEMS syndrome.

## Materials and methods

### Ethics

This study protocol was approved by the Institutional Review Board of Tangdu Hospital (No. K202404-04). This study involving human participants was in accordance with the ethical standards of the institutional and national research committee and with the 1964 Helsinki Declaration and its later amendments or comparable ethical standards. The data were collected on July 2024. We used an anonymized database for all analyses, and all potentially identifying variables were removed.

### Study subjects

The retrospective observational study included 19 patients with newly diagnosed POEMS syndrome between January 2018 and June 2024 in Tangdu Hospital. The diagnosis of POEMS syndrome was re-analyzed by Dr. Wen-Qing Wang, based on the updated criteria raised by Dispenzieri in 2023 (1). The mandatory major criteria are polyneuropathy (typically demyelinating) and monoclonal plasma cell-proliferative disorder (almost always λ). Three other major criteria are Castleman disease, sclerotic bone lesions, and VEGF elevation. Six minor criteria are organomegaly (splenomegaly, hepatomegaly, or lymphadenopathy), extravascular volume overload (edema, pleural effusion, or ascites), endocrinopathy (adrenal, thyroid, pituitary, gonadal, parathyroid, or panceratic), skin changes (hyperpigmantation, hypertrichosis, glomeruloid hemangiomata, plethora, acrocyanosis, flushing, or white nails), papilledema, thrombocytosis/polycythemia. The diagnosis of POEMS syndrome was based on the presentation of both mandatory major criteria, one of the three other major criteria, and one of the six minor criteria (1). The diagnosis criteria for each studied patient with POEMS syndrome are shown in Table 1. All patients were treatment-naïve, and did not suffer with important organ dysfunction. Meanwhile, twenty-five age- and sex-matched healthy individuals were also included as the control group.

**Table 1:** The diagnosis criteria each enrolled patients with POEMS syndrome.

**No**.	**Age (years)**	**Sex**	**Mandatory major criteria**		**Other major criteria**			**Minor criteria**					
			**Polyneuropathy**	**M-protein**	**Castlemandisease**	**Scleroticbone lesions**	**VEGFelevation** ^&^	**Organomegaly**	**Extravascular volume overload**	**Endocrinopathy**	**Skin changes**	**Papilledema**	**Thrombocytosis/ polycythemia**
1	38	F	Extremities numbness, electromyography: sensory-motor deficit	IgA (+) λ-LC (+)	–	–	1,335.6 pg/ml	Lymphadenopathy	Pericardial effusion	–	Hyperpigmentation	–	–
2	43	M	Extremities numbness, electromyography: sensory-motor deficit, demyelinating	IgG (+) λ-LC (+)	–	+	>800 pg/ml	Lymphadenopathy	–	Gonadal	Hyperpigmentation	+	–
3	39	M	Extremities numbness, electromyography: sensory-motor deficit, demyelinating	IgA (+) λ-LC (+)	–	+	550.2 pg/ml	Splenomegaly, lymphadenopathy	Pericardial and pleural effusion	–	Hyperpigmantation, hypertrichosis, white nails	–	–
4	71	F	Extremities numbness	IgA (+) λ-LC (+)	–	+	572.3 pg/ml	Splenomegaly, lymphadenopathy	Pleural effusion, ascites	Hypothyroidism	Hyperpigmantation	–	–
5	41	F	Extremities weakness, electromyography: sensory-motor deficit	+	–	–	828.2 pg/ml	Splenomegaly, lymphadenopathy	–	Hypothyroidism, panceratic	Hyperpigmantation, hypertrichosis, white nails	+	Platelets 572 × 10^9^/L
6	34	F	Extremities weakness, numbness, electromyography: sensory-motor deficit	IgA (+) λ-LC (+)	–	–	536.6 pg/ml	Lymphadenopathy	Pericardial and pleural effusion	Hypothyroidism	–	+	–
7	43	M	Extremities weakness, numbness, electromyography: sensory-motor deficit, demyelinating	IgG (+) λ-LC (+)	–	+	786.9 pg/ml	Lymphadenopathy	–	Hypothyroidism, panceratic	Hyperpigmantation	–	–
8	48	F	Extremities weakness, numbness	IgA (+) λ-LC (+)	–	+	252.0 pg/ml	Splenomegaly, hepatomegaly	Pericardial effusion, ascites	Gonadal	Hyperpigmantation	+	–
9	45	F	Extremities weakness, electromyography: sensory-motor deficit, demyelinating	IgA (+) λ-LC (+)	–	–	1.098.2 pg/ml	Splenomegaly, hepatomegaly	Edema, pericardial effusion, ascites	Hypothyroidism	Hyperpigmantation	–	–
10	60	F	Extremities numbness, electromyography: sensory-motor deficit, demyelinating	IgA (+) λ-LC (+)	–	+	17.25 pg/ml	Splenomegaly, hepatomegaly, lymphadenopathy	Edema, pericardial effusion, ascites	Hypothyroidism	Hyperpigmantation	–	–
11	47	M	Extremities numbness, neuropathic pain, electromyography: sensory-motor deficit	IgA (+) λ-LC (+)	–	+	Not determined	Splenomegaly, hepatomegaly, lymphadenopathy	Edema, pericardial effusion	Hypothyroidism, panceratic	Hyperpigmantation	–	–
12	36	M	Extremities weakness	IgA (+) λ-LC (+)	+	+	>800 pg/ml	Splenomegaly, hepatomegaly, lymphadenopathy	–	–	Hyperpigmantation	–	–
13	65	M	Extremities weakness, electromyography: sensory-motor deficit	IgA (+) λ-LC (+)	–	–	482.3 pg/ml	–	Edema, pericardial and pleural effusion, ascites	Hypothyroidism	Hyperpigmantation	–	–
14	49	F	Extremities weakness, electromyography: sensory-motor deficit, demyelinating	IgG (+) λ-LC (+)	–	+	1,061.2 pg/ml	Splenomegaly, hepatomegaly, lymphadenopathy	Pleural effusion, ascites	Hypothyroidism	Hyperpigmantation	–	–
15	52	M	Extremities weakness, numbness, electromyography: sensory-motor deficit, demyelinating	+	–	+	>1,600 pg/ml	Splenomegaly, hepatomegaly, lymphadenopathy, cardiomegaly	Edema, pericardial and pleural effusion, ascites	Hypoparathyroidism	Hyperpigmantation	–	–
16	50	M	Extremities numbness, electromyography: sensory-motor deficit, demyelinating	IgA (+) λ-LC (+)	–	–	>1,600 pg/ml	Splenomegaly, hepatomegaly, lymphadenopathy	Ascites	–	Hyperpigmantation	+	–
17	47	F	Extremities weakness, numbness, electromyography: sensory-motor deficit, demyelinating	IgG (+) λ-LC (+)	–	+	1,431.1 pg/ml	Splenomegaly, lymphadenopathy	Edema, pericardial and pleural effusion, ascites	Hypothyroidism	Hyperpigmantation	+	–
18	48	F	Extremities numbness, neuropathic pain, electromyography: sensory-motor deficit	IgG (+) κ-LC (+)	–	–	553.6 pg/ml	Cardiomegaly	Pericardial effusion	–	Hyperpigmantation	–	–
19	52	M	Extremities weakness, numbness, electromyography: sensory-motor deficit	IgG (+) λ-LC (+)	–	–	859.3 pg/ml	Splenomegaly, lymphadenopathy	Pleural effusion	–	Hyperpigmantation	–	–

F, female; M, male; λ-LC, λ-light chain; κ-LC, κ- light chain; VEGF, vascular endothelial growth factor.

^&^Normal range of VEGF: 0–142.2 pg/ml.

### Data collection

Clinical data from identified cases were abstracted from the medical records, including demographic information, underlying diseases, clinical manifestations, laboratory indicators when diagnosed as POEMS syndrome. The representative results of physical examination and ^18^fluorodeoxyglucose-positron emission tomography/computed tomoprahy (^18^F-FDG PET/CT) scanning as well as the therapeutic strategies were also collected.

### Statistical analyses

Statistical analyses were performed using SPSS Version 25.0 (IBM SPSS Software, Chicago, IL, USA). For categorical variables, data were presented as *n* (%). Fisher's exact test was used for comparison. Continuous variables following normal distribution were presented as mean ± standard deviation. Student's *t*-test was used for comparison between two groups. Paired *t*-test was used for comparison between patients before and post therapies. Continuous variables following skewed distribution were presented as median (interquartile range) [M (Q1, Q3)]. Mann-Whitney *U*-test was used for comparison between two groups. Wilcoxon matched pairs test was used for between patients before and post therapies. Spearman's rank-order correlation coefficient was used for determining the correlation between parameters for liver function and demographic or clinical index. A *P*-value < 0.05 was considered statistically significant.

## Results

### Baseline clinical and laboratory characteristics of patients with POEMS syndrome

**Figure 1:**
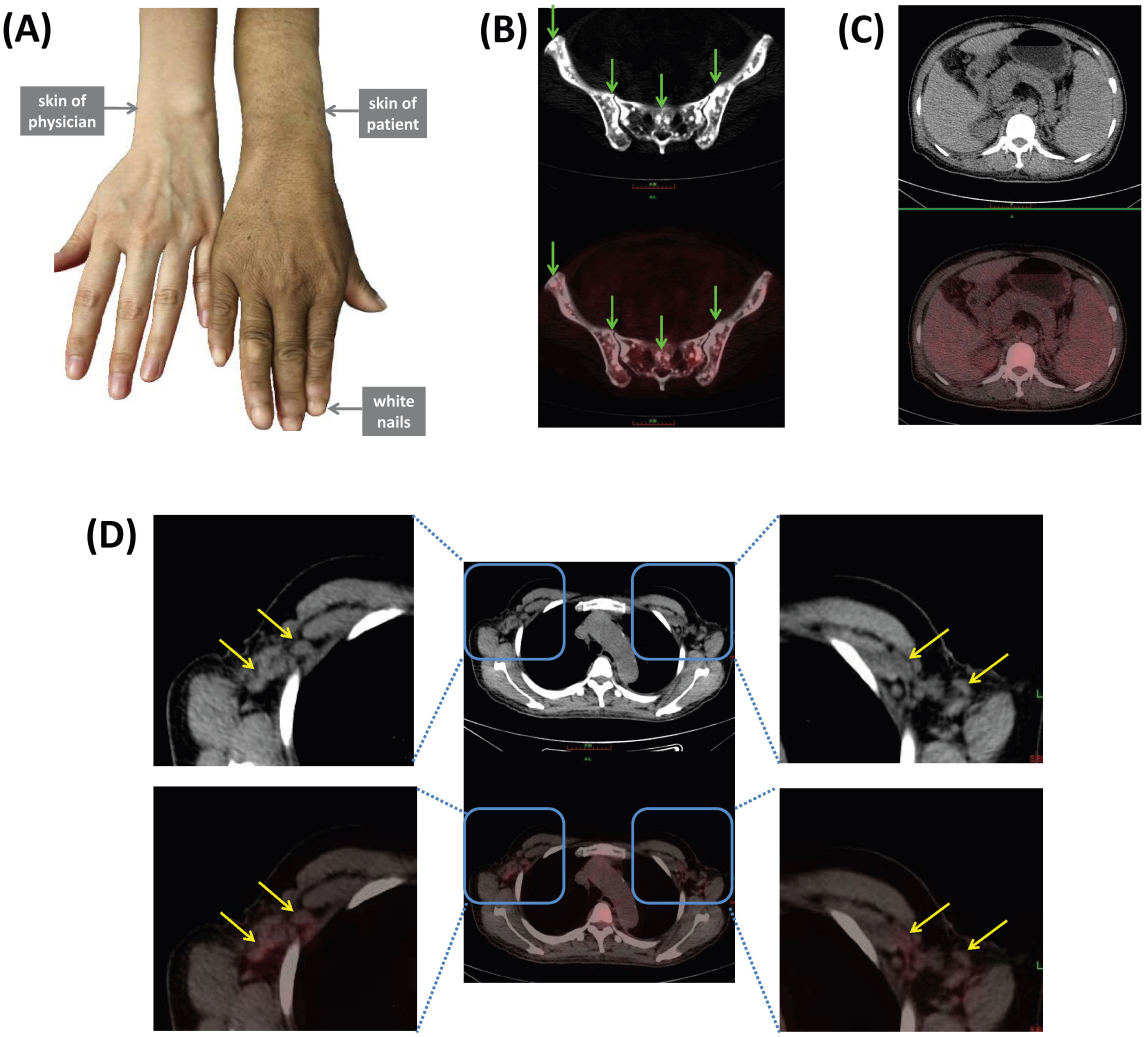
Representative manifestations of POEMS syndrome. **(A)** Skin changes including hyperpigmentation and white nails. ^18^F-FDG PET/CT scanning revealed **(B)** diffused osteosclerosis of pelvis (green arrows), **(C)** splenomegaly and hepatomegaly, **(D)** axillary fossa lymphadenectasis (yellow arrows).

All enrolled patients with POEMS syndrome matched diagnosis criteria updated in 2023 (Table 1). The first visit department was Neurology (initial symptoms: lower extremities weakness and numbness; *n* = 9), Infectious Diseases (initial symptoms: liver cirrhosis and ascites; *n* = 3), Nephrology (initial symptoms: edema; *n* = 3), Gastroenterology (initial symptoms: abdominal distension; *n* = 2), Rheumatology (initial symptoms: lower extremities numbness; *n* = 1), and Hematology (initial symptoms: lower extremities weakness; *n* = 1), respectively. All patients suffered with the symptoms of polyneuropathy. As previously reported, distal symmetric sensorimotor syndrome is the clinical presentation of polyneuropathy mostly frequently seen (27), which was found in all enrolled patients. Sensory, motor, or autonomic symptoms form the main focus depending on the type of nerve fiber involved (27). The sensory symptoms includes sensation of furriness and numbness, tingling, burning, and cold parasthesia, burning pain, stinging, electric shock-like pain, gait instability, falls (27). The main sensory symptoms of enrolled patients were extremities numbness (*n* = 14) and neuropathic pain (*n* = 2). The motor symptoms include weakness, muscle loss, muscle carmps, fasciculations (27). The main sensory symptom of enrolled patients was weakness (*n* = 11). Autonomic symptoms were dry skin, body hair loss, sensation of glare, bladder dysfunction, diarrhea, rapid heartbeat, gastrointestinal symptoms, urogenital symptoms (e.g., impaired micturitionerectile dysfunction) (27). However, no enrolled patients had obvious autonomic symptoms. Meanwhile, electromyography was performed in 16 patients, and the main presentations were sensory-motor deficit (*n* = 16) and demyelinating (*n* = 9). Furthermore, all patients were had monoclonal plasma cell-proliferative disorder, which presented as positive for serum M-protein. Two patients were positive for M-protein in serum protein electrophoresis, while 17 patients had were positive for M-protein in immunofixation electrophoresis, including 11 cases of IgA type λ-light chain (λ-LC), five cases of IgG type λ-LC, and one case of IgG type κ-LC. One case was confirmed as Castleman disease via lymph node biopsy. Eleven cases showed sclerotic bone lesions based on computed tomography (CT), magnetic resonance imaging (MRI), or ^18^F-FDG PET/CT scanning. Eighteen patients received serum VEGF quantification, and 17 of them had elevated serum VEGF level (>142.2 pg/ml). Organomegaly was found in 18 patients, which presented as lymphadenopathy (*n* = 15), splenomegaly (*n* = 13), hepatomegaly (*n* = 7), and cardiomegaly (*n* = 2). Extravascular volume overload was found in 15 patients, which presented as pericardial effusion (*n* = 10), pleural effusion (*n* = 8), ascites (*n* = 8), and edema (*n* = 5). The main presentation of endocrinopathy was hypothyroidism (*n* = 10), while the main presentation of skin changes was hyperpigmentation (*n* = 18) as the representative manifestation in Figure 1A. However, only one patient revealed thrombocytosis (platelets count 572 × 10^9^/L), and no patients manifested as polycythemia. Furthermore, six patients were also diagnosed as plasmacytoma, and two of them were multiple myeloma. Five patients had underlying chronic diseases, including chronic glomerulonephritis and liver cirrhosis. Importantly, four patients received ^18^F-FDG PET/CT scanning. The main findings in ^18^F-FDG PET-CT scanning were diffused osteosclerosis of pelvis (Figure 1B), splenomegaly and hepatomegaly (Figure 1C), axillary fossa lymphadenectasis (Figure 1D).

The baseline characteristics of enrolled subjects are shown in Table 2. There was no significant difference in white blood cell (WBC) count between patients with POEMS syndrome and controls (*P* = 0.547). Patients with POEMS syndrome had elevated platelet count (*P* = 0.002), while had reduced levels of red blood cell (RBC) count (*P* = 0.008) and hemoglobin (*P* < 0.001) compared with controls. Importantly, patients with POEMS syndrome had significant reduced levels of alanine aminotransferase (ALT), aspartate aminotransferase (AST), total bilirubin (T-BIL), total protein, and albumin compared with controls (all *P* < 0.05). Although there were no remarkable differences in urea nitrogen, creatinine, or eGFR between patients with POEMS syndrome and controls (*P* > 0.05), uric acid level was robustly elevated in patients with POEMS syndrome (*P* = 0.003). Patients with POEMS syndrome had significantly down-regulated Na^+^ and Ca^2+^ levels (*P* < 0.001) and up-regulated blood glucose level (*P* = 0.007). Patients with POEMS syndrome had reduced triiodothyronine (T3), thyroxine (T4), free triiodothyronine (FT3), and free thyroxine (FT4) levels and elevated thyroid stimulating hormone (TSH) level due to the diagnosis of hypothyroidism in ten patients (*P* < 0.001). The levels of immunoglobulin and complement were only assessed in patients with POEMS syndrome, and λ-light chain (λ-LC) level was slightly higher than the upper limit of normal (normal range: 0.9–2.1 g/L).

**Table 2:** Baseline characteristics of enrolled subjects.

	**POEMS syndrome**	**Controls**	***P*-value**
Case (*n*)	19	25	–
Age (years)	47.89 ± 9.57	45.24 ± 5.64	0.256
Male gender (*n*, %)	9 (47%)	13 (52%)	0.761
Female gender (*n*, %)	10 (53%)	12 (48%)	0.761
**Blood routine test**			
WBC (× 10^9^/L)	5.94 ± 1.99	5.65 ± 1.21	0.547
Platelet (× 10^9^/L)	318.9 ± 97.72	241.6 ± 53.87	0.002
RBC (× 10^12^/L)	4.35 ± 0.62	4.78 ± 0.38	0.008
Hemoglobin (g/L)	127.1 ± 13.41	145.8 ± 14.70	< 0.001
**Liver function**			
ALT (U/L)	11 (6, 18)	16 (11, 27)	0.016
AST (U/L)	12 (10, 16)	21 (18, 24)	< 0.001
T-BIL (μmol/L)	11.26 ± 4.03	17.66 ± 5.85	< 0.001
Total protein (g/L)	61.25 ± 4.85	72.98 ± 4.15	< 0.001
Albumin (g/L)	35.11 ± 4.01	45.42 ± 2.89	< 0.001
Globulin (g/L)	26.13 ± 5.02	27.56 ± 3.41	0.269
**Renal function**			
Urea nitrogen (mmol/L)	5.30 (4.21, 11.80)	5.40 (4.10, 7.05)	0.507
Creatinine (μmol/L)	66.00 (53.20, 100.0)	70.00 (54.00, 80.00)	0.981
Uric acid (μmol/L)	428.4 ± 142.2	327.9 ± 65.29	0.003
eGFR (ml/min/1.73 m^2^)	115.0 (58.84, 147.2)	101.2 (93.01, 117.0)	0.840
**Electrolyte**			
K^+^ (mmol/L)	4.46 ± 0.87	4.37 ± 0.25	0.622
Na^+^ (mmol/L)	138.1 ± 3.05	140.9 ± 1.70	< 0.001
Cl^−^ (mmol/L)	105.1 ± 2.77	104.2 ± 1.72	0.189
Ca^2+^ (mmol/L)	2.12 ± 0.14	2.38 ± 0.10	< 0.001
Blood glucose (mmol/L)	5.89 ± 1.36	5.07 ± 0.44	0.007
**Thyroid function**			
T3 (nmol/L)	1.03 (0.69, 1.60)	1.79 (1.58, 2.08)	< 0.001
T4 (nmol/L)	75.05 ± 22.25	102.4 ± 15.63	< 0.001
FT3 (pmol/L)	2.98 ± 1.31	4.82 ± 0.82	< 0.001
FT4 (pmol/L)	11.92 ± 3.39	18.03 ± 2.32	< 0.001
TSH (uIU/mL)	7.71 (4.98, 12.56)	1.62 (1.22, 3.13)	< 0.001
**Immunoglobulin (Ig) and complement (C)**			
IgG (g/L)	12.00 (9.27, 13.70)	N.D.	–
IgA (g/L)	3.57 (1.85, 7.14)	N.D.	–
IgM (g/L)	0.88 (0.46, 1.53)	N.D.	–
κ-LC (g/L)	2.37 (1.99, 3.05)	N.D.	–
λ-LC (g/L)	2.25 (1.79, 2.50)	N.D.	–
κ/λ	1.07 (0.95, 1.38)	N.D.	–
C3 split product (g/L)	0.92 ± 0.16	N.D.	–
C4 (g/L)	0.24 ± 0.06	N.D.	–
C1 inhibitor (g/L)	0.30 ± 0.08	N.D.	–

WBC, white blood cells; RBC, red blood cells; ALT, alanine aminotransferase; AST, aspartate aminotransferase; T-BIL, total bilirubin; eGFR, estimated glomerular filtration rate; T3, triiodothyronine; T4, thyroxine; FT3, free triiodothyronine; FT4, free thyroxine; TSH, thyroid stimulating hormone; κ-LC, κ-light chain; λ-LC, λ- light chain; N.D., not determined.

### Correlations between clinical indexes in patients with POEMS syndrome

The correlations between clinical indexes in patients with POEMS syndrome were analyzed, and the results are shown in Table 3. There were several common correlations between clinical indicators. Serum K^+^ level was positively correlated with urea nitrogen, creatinine, and uric acid levels, while negatively correlated with eGFR (all *P* < 0.05), because potassium are mainly excreted through urine. Serum κ-LC level was positively correlated with levels of IgG (*r* = 0.60, *P* = 0.007) and IgM (*r* = 0.49, *P* = 0.032), while serum λ-LC level was positively correlated with IgA level (*r* = 0.70, *P* < 0.001) because most of the patients (58%, 11/19) had IgA type λ-LC M-protein based on immunofixation electrophoresis analysis. Importantly, serum T3, T4, and FT3 levels were positively correlated with hemoglobin and eGFR, while negatively correlated with urea nitrogen, creatinine, and uric acid levels (all *P* < 0.05), indicating the correlation between reduced thyroid function and impaired renal function. Furthermore, serum VEGF level was negatively correlated with Ca^2+^ level in patients with POEMS syndrome (*r* = −0.56, *P* = 0.016).

**Table 3:** The correlations between clinical indexes in patients with POEMS syndrome.

	**Blood routine test**				**Liver function**							**Renal function**			**Electrolyte**				**BG**	**Thyroid function**					**Immunoglobulin (Ig) and complement (C)**								**VEGF**
	**WBC**	**PLT**	**RBC**	**HGB**	**ALT**	**AST**	**T-BIL**	**TP**	**ALB**	**GLO**	**BUN**	**Cr**	**UA**	**eGFR**	**K** ^+^	**Na** ^+^	**Cl** ^−^	**Ca** ^2+^		**T3**	**T4**	**FT3**	**FT4**	**TSH**	**IgG**	**IgA**	**IgM**	κ**-LC**	λ**-LC**	**C3SP**	**C4**	**C1I**	
WBC		0.26	0.31	0.14	0.24	0.42	−0.35	0.08	0.14	−0.03	0.07	015	−0.22	−0.01	0.04	0.42	0.05	0.02	0.29	0.16	0.03	0.01	−0.08	−0.11	−0.24	−0.07	0.04	0.22	−0.45	0.40	0.27	0.40	0.03
PLT			0.01	−0.09	−0.10	−0.10	−0.16	**−**0.64 ^**#**^	−0.27	−0.41	−0.10	−0.02	−0.16	0.13	−0.30	0.23	−0.01	−0.16	0.25	0.02	0.22	0.08	0.04	−0.24	−0.20	−0.02	−0.03	−0.04	−0.23	0.24	−0.01	0.13	0.07
RBC				0.84 ^**#**^	−0.05	0.20	−0.19	0.25	0.37	−0.05	0.05	−0.18	−0.12	0.36	0.12	0.11	–0.49^**#**^	0.08	0.47 ^**#**^	0.60 ^**#**^	0.44	0.44	0.34	−0.22	−0.12	−0.13	−0.24	−0.01	−0.24	0.40	0.37	0.25	0.16
HGB					−0.06	0.08	0.18	0.23	0.39	−0.10	−0.10	−0.30	−0.23	0.46	−0.03	0.25	−0.43	0.28	0.36	0.76 ^**#**^	0.59 ^**#**^	0.62 ^**#**^	0.45	0.01	−0.01	−0.22	−0.12	−0.10	−0.16	0.33	0.32	−0.17	0.03
ALT						0.74 ^**#**^	−0.00	0.09	−0.02	0.30	−0.37	0.20	0.17	−0.20	0.22	0.04	−0.27	0.20	−0.03	−0.35	**−**0.52 ^**#**^	−0.46	−0.39	0.19	−0.18	−0.00	−0.03	−0.06	−0.14	0.14	−0.06	0.11	−0.21
AST							−0.33	0.31	0.02	0.38	0.33	0.25	−0.01	−0.13	0.30	0.09	−0.18	0.22	−0.13	−0.17	−0.34	−0.29	−0.30	0.20	−0.28	0.30	0.22	0.30	−0.13	0.08	−0.11	0.41	−0.38
T–BIL								0.02	−0.11	0.11	−0.29	−0.20	−0.14	0.07	−0.38	0.01	−0.16	0.09	−0.14	0.24	0.11	−0.05	0.05	0.65 ^**#**^	0.31	−0.28	−0.13	−0.37	0.18	0.26	0.27	−0.40	0.07
TP									0.37	0.67 ^**#**^	−0.20	−0.27	−0.25	0.23	−0.08	0.02	−0.31	0.20	−0.19	0.26	0.19	0.02	0.06	0.48	0.37	0.06	0.13	0.39	0.23	−0.01	0.15	−0.23	−0.11
ALB										−0.44	−0.01	−0.17	−0.20	0.27	0.23	0.22	0.05	0.48 ^**#**^	0.18	0.40	0.27	0.44	0.27	−0.26	−0.31	−0.19	0.25	0.07	**−**0.48 ^**#**^	0.07	0.14	−0.17	0.02
GLO											−0.11	−0.04	−0.09	−0.08	−0.26	−0.15	−0.34	−0.19	−0.33	−0.09	−0.03	−0.34	−0.17	0.73 ^**#**^	0.61 ^**#**^	0.22	−0.07	0.32	0.60 ^**#**^	−0.06	0.01	−0.06	−0.02
BUN												0.84 ^**#**^	0.82 ^**#**^	**−**0.72 ^**#**^	0.82 ^**#**^	−0.14	0.01	0.07	−0.02	**−**0.61 ^**#**^	**−**0.68 ^**#**^	**−**0.64 ^**#**^	−0.40	0.01	**−**0.52 ^**#**^	0.36	−0.18	−0.21	0.06	−0.04	−0.06	0.55 ^**#**^	−0.11
Cr													0.72 ^**#**^	**−**0.68 ^**#**^	0.78 ^**#**^	−0.06	0.23	−0.06	−0.32	**−**0.67 ^**#**^	**−**0.67 ^**#**^	**−**0.60 ^**#**^	−0.44	0.19	−0.37	0.35	−0.16	−0.08	0.10	0.10	0.10	0.75 ^**#**^	−0.15
UA														**−**0.95 ^**#**^	0.60 ^**#**^	**−**0.57 ^**#**^	−0.02	−0.28	0.05	**−**0.66 ^**#**^	**−**0.66 ^**#**^	**−**0.72 ^**#**^	−0.49	−0.00	−0.43	0.50 ^**#**^	−0.36	−0.33	0.27	−0.18	0.03	0.44	0.01
eGFR															**−**0.63 ^**#**^	0.17	−0.23	0.18	0.40	0.74 ^**#**^	0.66 ^**#**^	0.80 ^**#**^	0.60 ^**#**^	−0.30	0.23	**−**0.31	0.21	0.12	−0.25	−0.12	−0.16	**−**0.67 ^**#**^	0.11
K^+^																−0.43	0.29	0.05	0.22	0.11	−0.30	−0.29	−0.04	−0.02	−0.32	0.10	−0.15	−0.02	−0.19	−0.07	−0.06	0.48	−0.11
Na^+^																	0.29	−0.01	−0.06	0.28	0.21	0.30	−0.19	0.26	0.18	−0.25	0.68 ^**#**^	0.38	−0.22	−0.08	−0.11	−0.30	0.12
Cl^−^																		0.03	−0.09	−0.34	−0.20	−0.13	−0.13	−0.06	0.10	−0.23	0.45	0.26	−0.22	**−**0.55 ^**#**^	**−**0.49 ^**#**^	−0.04	−0.04
Ca^2+^																			0.17	−0.04	0.39	0.37	0.54 ^**#**^	0.02	−0.19	−0.10	0.13	−0.01	−0.29	007	0.14	−0.16	**−**0.56 ^**#**^
BG																				0.32	0.17	0.33	0.40	**−**0.60 ^**#**^	−0.24	−0.25	−0.39	−0.20	−0.43	0.18	0.31	0.25	0.11
T3																					0.78 ^**#**^	0.94 ^**#**^	0.58 ^**#**^	−0.24	0.14	−0.32	0.27	0.09	−0.27	0.38	0.45	−0.40	0.47
T4																						0.77 ^**#**^	0.83 ^**#**^	−0.02	0.26	−0.51	0.23	0.25	−0.29	0.27	0.35	−0.19	0.20
FT3																							0.72 ^**#**^	−0.38	−0.04	−0.47	0.15	−0.02	−0.36	0.26	0.33	−0.15	0.17
FT4																								−0.31	−0.04	**−**0.54 ^**#**^	−0.11	−0.15	−0.38	−0.25	0.23	−0.07	0.02
TSH																									0.31	0.42	0.18	0.19	0.69 ^**#**^	0.16	0.10	−0.01	−0.28
IgG																										−0.32	0.18	0.60 ^**#**^	0.34	−0.27	−0.08	−0.43	0.25
IgA																											−0.12	−0.10	0.70 ^**#**^	0.05	0.02	0.36	−0.25
IgM																												0.49 ^**#**^	−0.09	−0.46	−0.33	−0.26	0.02
κ-LC																													0.02	−0.36	−0.07	−0.18	0.06
λ-LC																														−0.15	−0.12	−0.08	−0.09
C3SP																															0.66 ^**#**^	0.28	0.08
C4																																0.33	0.16
C1I																																	−0.17
VEGF																																	

WBC, white blood cells; PLT, platelets; RBC, red blood cells; HGB, hemoglobin; ALT, alanine aminotransferase; AST, aspartate aminotransferase; T-BIL, total bilirubin; TP, total protein; ALB, albumin; GLO, globulin; BUN, urea nitrogen; Cr, creatinine; UA, uric acid; eGFR, estimated glomerular filtration rate; BG, blood glucose; T3, triiodothyronine; T4, thyroxine; FT3, free triiodothyronine; FT4, free thyroxine; TSH, thyroid stimulating hormone; κ-LC, κ-light chain; λ-LC, λ- light chain; C3SP, C3 split product; C1I, C1 inhibitor.

^#^*P* < 0.05.

The bold and underline values with # indicate statistical correlations with *P* value less than 0.05.

### Changes of laboratory indexes in response to therapy in patients with POEMS syndrome

Fourteen patients with POEMS syndrome received bortezomib/ixazomib and lenalidomide/thalidomide therapy, and two of them received hematopoietic stem cell transplantation. The patients were followed-up at least 6 months post-therapy. The symptoms of polyneuropathy (especially distal symmetric sensorimotor syndrome) as well as the hyperpigmentation of the skin were ameliorated in response to therapy. VEGF level was reduced in response to therapy in eleven patients, but three patients had increased serum VEGF level post-therapy. Routine tests, including blood routine test, liver and renal function, and electrolytes, were performed in those fourteen patients before and post therapy. Platelet count was robustly down-regulated to the normal range in response to therapy (*P* = 0.001, Table 4). However, there were no significant differences in WBC, RBC, or hemoglobin level between baseline and post-therapy (all *P* > 0.05, Table 4). AST and total protein levels were increased in response to therapy (all *P* < 0.05, Table 4). Meanwhile, ALT and albumin levels were also increased post-therapy, however, this difference just failed to achieve statistical significance (*P* = 0.090 and *P* = 0.069, Table 4). Serum creatinine and uric acid levels were reduced, while eGFR level was increased post-therapy (all *P* < 0.05, Table 4). There were no remarkable differences in urea nitrogen or electrolytes levels between baseline and post-therapy (all *P* > 0.05, Table 4). Taken together, effective therapeutic strategies normalized the platelet count, AST and total protein level, as well as the main kidney functions, especially eGFR.

**Table 4:** Clinical indexes in patients with POEMS syndrome in response to therapy (*n* = 14).

	**Baseline**	**Post-therapy**	***P*-value**
**Blood routine test**			
WBC (× 10^9^/L)	6.36 ± 1.95	6.07 ± 3.23	0.748
Platelet (× 10^9^/L)	328.0 ± 108.5	211.3 ± 121.3	0.001
RBC (× 10^12^/L)	4.24 ± 0.59	4.22 ± 0.89	0.951
Hemoglobin (g/L)	124.1 ± 13.57	123.7 ± 29.44	0.955
**Liver function**			
ALT (U/L)	11 (5, 18)	16 (12, 29)	0.090
AST (U/L)	12 (9, 16)	18 (13, 24)	0.006
T-BIL (μmol/L)	11.42 ± 4.50	16.95 ± 9.78	0.029
Total protein (g/L)	61.35 ± 4.38	65.40 ± 10.20	0.145
Albumin (g/L)	34.97 ± 3.89	38.98 ± 6.66	0.069
Globulin (g/L)	26.36 ± 5.14	26.44 ± 7.35	0.961
**Renal function**			
Urea nitrogen (mmol/L)	5.10 (4.21, 12.43)	6.25 (4.60, 9.77)	0.903
Creatinine (μmol/L)	68.50 (57.40, 103.8)	62.00 (51.25, 72.60)	0.030
Uric acid (μmol/L)	424.6 ± 134.7	312.1 ± 140.0	0.026
eGFR (ml/min/1.73 m^2^)	88.18 (57.77, 134.2)	114.3 (75.26, 157.6)	0.027
**Electrolyte**			
K^+^ (mmol/L)	4.46 ± 1.16	4.16 ± 0.43	0.366
Na^+^ (mmol/L)	138.7 ± 3.84	139.2 ± 4.77	0.746
Cl^−^ (mmol/L)	106.1 ± 2.61	105.1 ± 3.62	0.483
Ca^2+^ (mmol/L)	2.05 ± 0.14	2.17 ± 0.26	0.207

WBC, white blood cells; RBC, red blood cells; ALT, alanine aminotransferase; AST, aspartate aminotransferase; T-BIL, total bilirubin; eGFR, estimated glomerular filtration rate.

## Discussion

In this retrospective study, we reported the clinical characteristics of patients with POEMS syndrome in a tertiary-care hospital in Xi'an, China. We found that the most common symptoms of initial symptoms were lower extremities weakness and numbness (58%, 11/19), followed by the symptoms of extravascular volume overload (32%, 6/19). There were several abnormal routine laboratory indexes in newly diagnosed POEMS syndromes, including the up-regulation of platelet count, uric acid, and λ-LC level as well as the down-regulation of RBC count, hemoglobin, total protein, albumin, Ca^2+^, and thyroid hormone levels. Furthermore, although there were statistical differences in ALT, AST, T-BIL, Na^+^, and blood glucose levels between patients with POEMS syndrome and healthy individuals, these indicators were still within the normal range. Therefore, the clinical manifestations of POEMS syndrome were complex and diverse, with significant individual differences.

The diagnosis of POEMS syndrome was based on the two mandatory major criteria, three major criteria, and six minor features (1, 28, 29). Except for the presentation of polyneuropathy and positive for M-protein in all 19 patients, we found that elevated VEGF (17 cases), organomegaly (18 cases), and hyperpigmentation (18 cases) were the most common criteria for diagnosis of POEMS syndrome. We also found that elevated VEGF was negatively correlated with serum Ca^2+^ level, which was reduced in patients with POEMS syndrome. This was consistent with the physiological finding that VEGF could stimulate calcium ion flux in endothelial cells (30), mediating the elevation of intracellular release and extracellular entry of calcium (31, 32). In turn, the intracellular calcium could also regulated VEGF secretion in human adipocytes. The serum Ca^2+^ level indicated the extracellular calcium, which might reflect the elevation of intracellular calcium, resulting in the elevation of VEGF and vascular permeability (32, 33). Thus, VEGF calcium signaling in endothelial cells (33) might contribute to the pathogenesis and disease progression of POEMS syndrome. Moreover, ^18^F-FDG PET/CT is a useful tool for diagnosis and evaluation of patients with POEMS syndrome by providing the evidence of bone lesions, liver or spleen enlargement, lymphadenopathy, and cavity effusion (34). Four patients received ^18^F-FDG PET/CT scanning, and the systemic findings were found in these patients, including the osteosclerosis (normometabolic bone changes), lymphadenopathy, hepatomegaly, and splenomegaly. Finally, although thrombocytosis/polycythemia has been involved in the updated diagnostic criteria of six minor features, we found slightly elevation of platelet count and only one patient match the diagnosis of thrombocytosis (platelet count > 450 × 10^9^/L). However, the mean hemoglobin level was low (127.1 g/L) and nine patients (47%) suffered with mild anemia. This was consistent with the report on consecutive Chinese cohort, showing that 26% patients had anemia (35). In our opinion, impaired renal function and difference in studied race might contribute to the anemia, which were still needed for further investigation.

The liver and renal function was not involved in the diagnostic criteria for POEMS syndrome, but these indicators were most commonly used in clinical practice for evaluation the status of patients. It was well-accepted that low serum albumin and reduced eGFR was poor prognostic risk factor for POEMS syndrome (1, 21, 28, 29). Herein, we found several abnormal indicators corresponding to liver and renal function in POEMS syndrome. Although ALT, AST, and T-BIL levels were within the normal range, these indicators were robustly down-regulated compared with healthy individuals. Furthermore, effective risk-adapted therapy increased the ALT, AST, and T-BIL levels. However, ALT, AST, and T-BIL levels did not strongly correlate with other clinical indicators in patients with POEMS syndrome. The elevations of aminotransferases reflected the hepatocytes damage and liver toxicity in several disease conditions. However, even the three patients with liver cirrhosis had very low levels of ALT (11, 14, and 20 U/L), AST (12, 13, and 14 U/L), and T-BIL (15.3, 7.6, and 9.4 μmol/L). The cause of decreased blood aminotransferase activity included drug-induced and metabolic categories (36). On the one hand, several patients had underlying diseases, but the administration of drugs did not contain the agents causing decreases in aminotransferase activity, such as delapril hydrochloride, izoniazid, cefazolin, canavanine, or oxodipine (36). On the other hand, metabolic factors, including severe weight loss, anorexia, vitamin B6 or zinc deficiency, also contributed to ALT and AST reduction (36). We did not assess the vitamin B6 or zinc level in patients with POEMS syndrome, but we still assumed that metabolic factors might play essential roles in decreased ALT, AST, and T-BIL levels in POEMS syndrome. Furthermore, Kourelis et al. indicated that albumin > 32 g/L was one of the important factors which were associated with superior overall survival of POEMS syndrome (21). We found that albumin level was reduced, and effective therapy increased the albumin level in patients with POEMS syndrome. Moreover, serum λ-LC was negatively correlated with albumin and positively correlated with globulin in patients with POEMS syndrome. Therefore, the quantification of λ-LC might also be associated with disease progression and outcomes of POEMS syndrome.

Renal impairment was a common complication of POEMS syndrome, and might be independently associated with ascites (37). In the current study, we found that eight patients (42%) with POEMS syndrome had reduced eGFR level (< 90 ml/min/1.73 m^2^), including five moderate impairment (eGFR 30–59 ml/min/1.73 m^2^) and three severe dysfunction (eGFR < 30 ml/min/1.73 m^2^), which was consistent with the previous findings (37). However, the median level of eGFR was comparable with healthy individuals. Meanwhile, uric acid level was increased in POEMS syndrome. Importantly, effective therapy reversed the renal impairment, which presented as down-regulation of creatinine and uric acid as well as up-regulation of eGFR. Importantly, the impaired renal function seemed to be strongly associated with hypothyroidism in POEMS syndrome. Previous studies revealed that subclinical hypothyroidism independently correlated with poor renal outcomes in patients with chronic kidney disease (38), and levothyroxine replacement therapy improved renal function in hypothyroidism patients (39). There might be a complex interaction between thyroid hormone and renal function. Although the several patients with POEMS syndrome complicated received levothyroxine replacement administration, they were not followed-up for thyroid function. Thus, we could not analyze the dynamic association between renal and thyroid function in POEMS syndrome.

There were several limitations in this study. Firstly, due to the very low incidence of POEMS syndrome and limited sample size, the expanded sample size through multi-center collaboration should be performed to strengthen the validity of the current conclusions. Secondly, we only tracked changes within 6 months post-treatment, missing long-term prognosis and outcomes. This was due to the fact that most patients were enrolled between the year 2023 and 2024. Extending the follow-up period would provide more comprehensive insights into treatment efficacy and quality of life. Thirdly, although we found the correlation between VEGF elevation and calcium signaling, further experimental studies exploring the pathophysiology of POEMS syndrome, leading to the in-depth investigation into the underlying mechanisms. Precise medication and deep learning might contribute to POEMS syndrome pathogenesis (40).

In summary, several patients with POEMS syndrome had impaired liver and renal function, and effective therapy might partly repair the liver and renal dysfunction. It is important for early diagnosis of POEMS syndrome, especially for general and neurological physicians. For those patients who suffered with polyneuropathy, the common and important laboratory indicators (blood routine test, liver and renal functions, electrolyte, thyroid function, etc.) should be monitors for early recognition of POEMS syndrome.

## Data Availability

The original contributions presented in the study are included in the article/supplementary material, further inquiries can be directed to the corresponding author.
